# Computational data mining method for isotopomer analysis in the quantitative assessment of metabolic reprogramming

**DOI:** 10.1038/s41598-019-57146-8

**Published:** 2020-01-14

**Authors:** Fumio Matsuda, Kousuke Maeda, Nobuyuki Okahashi

**Affiliations:** Department of Bioinformatic Engineering, Graduate School of Information Science and Technology, Osaka University, Osaka, Japan

**Keywords:** Metabolomics, Analytical biochemistry

## Abstract

Measurement of metabolic flux levels using stable isotope labeling has been successfully used to investigate metabolic redirection and reprogramming in living cells or tissues. The metabolic flux ratio between two reactions can be estimated from the ^13^C-labeling patterns of a few metabolites combined with the knowledge of atom mapping in the complicated metabolic network. However, it remains unclear whether an observed change in the labeling pattern of the metabolites is sufficient evidence of a shift in flux ratio between two metabolic states. In this study, a data analysis method was developed for the quantitative assessment of metabolic reprogramming. The Metropolis-Hastings algorithm was used with an *in silico* metabolic model to generate a probability distribution of metabolic flux levels under a condition in which the ^13^C-labeling pattern was observed. Reanalysis of literature data demonstrated that the developed method enables analysis of metabolic redirection using whole ^13^C-labeling pattern data. Quantitative assessment by Cohen’s effect size (*d*) enables a more detailed read-out of metabolic reprogramming information. The developed method will enable future applications of the metabolic isotopomer analysis to various targets, including cultured cells, whole tissues, and organs.

## Introduction

Measurement of metabolic flux levels has been successfully used to investigate metabolic reprogramming or redirection in living cells or tissues of microbes, plants, and mammals^1–5^. A series of flux analysis methodologies based on stable isotope labeling have been used for the comparison of metabolic flux distribution in distinct metabolic states^6–13^. ^13^C-metabolic flux analysis (^13^C-MFA) is one of the most quantitative and sophisticated methods available to investigate flux distribution in the central carbon metabolism. There are several requirements for ^13^C-MFA experiments (Requirement 1–5), as listed in Table 1 ^14–20^.

**Table 1:** Comparison of methodologies for metabolic flux analysis.

Requirements	^13^C-metabolic flux analysis (^13^C-MFA)	Isotopomer analysis (Conventional)	Isotopomer analysis (This study)
1. (Pseudo) metabolic and isotopically steady state	Required	Required	Required
2. Specific rates data	Required	Not required	Not required
3. *In silico* metabolic model	Required for finding best-fitted metabolic flux	Not required	Required for the Metropolis-Hastings algorithm
4. Number of data points	Larger than degree of freedom of *in silico* metabolic model	No restriction	Larger number is preferable
5. Statistical estimation of significance	95% confidence interval of point estimation result	Not evaluated	Cohen’s effect size, *d*

**Figure 1:**
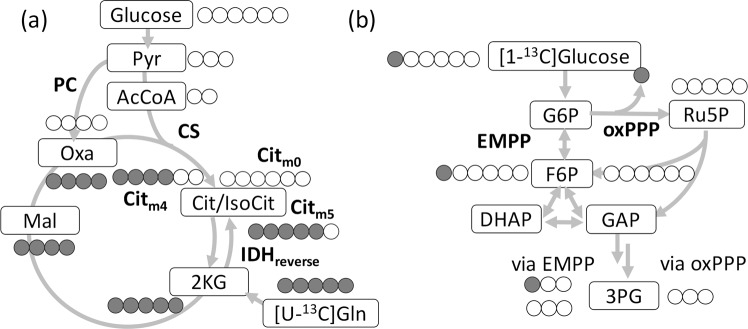
Example of atom mappings of the ^13^C-labeling experiment used for isotopomer analysis. (**a**) Simplified metabolic pathways related to the reductive glutamine metabolism including citrate (Cit) biosynthesis from [U-^13^C]glutamine (Gln) and non-labeled from glucose. (**b**) Simplified metabolic pathways related to the oxidative pentose phosphate pathway (oxPPP) and the Embden–Meyerhof–Parnas pathway (EMPP) from [1-^13^C]glucose. Filled and open circles represent ^13^C and ^12^C atoms respectively. CS: citrate synthase, PC: pyruvate carboxylase, IDH_reverse_: reverse reaction of isocitrate dehydrogenase. All abbreviations are described in Supplementary Table S1.

Recently, a more simplified method termed flux analysis or isotopomer analysis has been used for the mammalian cell analysis. The original methodology for the isotopomer-based analysis of local flux ratios was developed more than decades ago^21^. It enables the qualitative survey of metabolic redirections that occur in cells, such as cancer and immune cells^22–28^. A metabolic flux ratio between two pathways can be estimated from the ^13^C-labeling patterns or the mass isotopomer distribution vector (MDV) of the specific metabolites measured by mass spectrometry^29,30^. Isotopomer analysis has been often employed to investigate the reductive glutamine metabolism or the reverse reaction of isocitrate dehydrogenase (IDH_reverse_) in cancer cells^31–33^ (Fig. 1a). The labeling patterns of citrate (Cit) have been measured in cells cultured in a medium containing [U-^13^C]glutamine. This measurement is possible because the citrate molecule labeled with five ^13^C atoms (Cit_m5_) is derived from IDH_reverse_ or the reductive glutamine metabolism (Fig. 1a). On the other hand, the clockwise reaction of tricarboxylic acid (TCA) cycle produces Cit_m4_ via citrate synthase (CS). All abbreviations used in this study are described in Supplementary Table S1.

Similarly, the medium containing [1,2-^13^C]glucose or [1-^13^C]glucose has been used to estimate the metabolic flux ratio between the Embden–Meyerhof–Parnas pathway (EMPP) and the oxidative pentose phosphate pathway (oxPPP)^34–36^. From [1-^13^C]glucose, the EMPP generates single ^13^C-labeled intermediates of glycolysis, such as [1-^13^C]fructose 6-phosphate (F6P) and [3-^13^C]3-phosphoglycerate (3PG). The oxPPP supplies non-labeled intermediates, as the ^13^C atom is metabolically discarded as ^13^CO_2_. Thus, the relative abundances of the ^13^C-labeled and non-labeled intermediates could help estimate the metabolic flux ratio between the EMPP and oxPPP^37,38^.

Isotopomer analysis has been widely applied for many metabolism studies because of its simpler experimental design (Table 1)^29^. In most studies, exponential growth phase cells have been used to measure MDVs of intracellular metabolites to ensure (pseudo) metabolic and isotopically steady states (Requirement 1 in Table 1). An interpretation of the results of the isotopomer analysis remains puzzling as the relationship between an observed change in labeling patterns and metabolic redirection is not straightforward. The labeling of Cit by [U-^13^C]glutamine can be affected by the rearrangement of the carbon skeleton by anaplerotic reactions including pyruvate carboxylase (PC) (Fig. 1a). The reversible reactions in the upper part of the EMPP and the PPP can also cause a complex rearrangement of the carbon skeleton (Fig. 1b). The complex labeling nature hinders a clear understanding of whether a measured change in the labeling patterns provides definitive evidence to support the metabolic redirection of interest. For the same reason, the occurrence of other isotopomers has been ignored in isotopomer analysis, although the labeling pattern data reflect the metabolic flux information of metabolic pathways. Recently, an analysis of metabolic changes in bacterial systems using ^13^C-labeling pattern data without complete ^13^C-MFA has been attempted by PCA analyses and isotopomer comparisons^39^.

In order to extend the isotopomer analysis, a computational method using an *in silico* metabolic model (Requirement 3) was developed in this study to analyze data obtained by the isotopomer analysis. The method enabled the quantitative assessment of whether an observed change in a labeling pattern of metabolites was sufficient evidence of a shift of a flux ratio between two metabolite states based on the Cohen’s effect size (*d*) (Requirement 5). Moreover, reanalysis of literature data demonstrated that the developed method provided more detailed information concerning metabolic redirection.

## Results

### Design of computational data analysis method for isotopomer analysis

**Figure 2:**
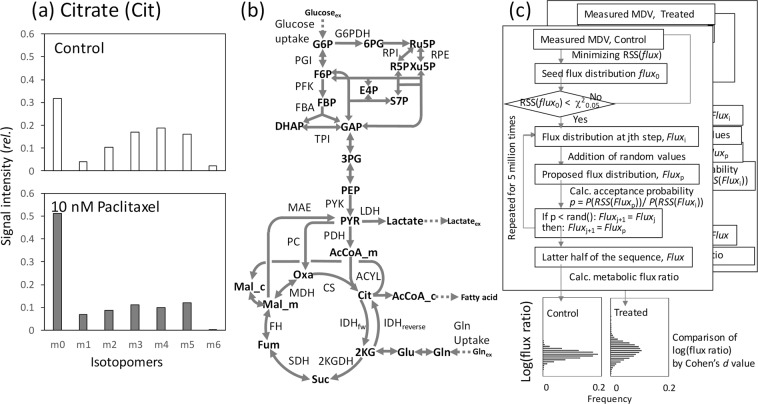
Data analysis method proposed in this study. (**a**) Example isotope labeling data of citrate obtained from MCF-7 breast cancer cells cultured in a medium containing non-labeled glucose and [U-^13^C]glutamine. Data were obtained from untreated cells or cells treated with paclitaxel (an anti-cancer drug) for 24 h. The effect of the natural isotopes was removed. The data are derived from our previous ^13^C-metabolic flux analysis study^40^. (**b**) The metabolic model used in this study. The model includes 53 reactions and 34 metabolites. Forward and reverse reactions of isocitrate dehydrogenase (IDH) are specifically described in the figure. All abbreviations are shown in Supplementary Table S1. (**c**) Procedure developed for the data analysis. A seed metabolic flux distribution, *flux*, was produced by minimizing the RSS(*flux*) for the measured mass spectra (MDV) data and was used as the 0th metabolic flux distribution, *flux*_0_, when an over-fitting result was obtained. Based on a flux distribution at step j, *flux*_j_, a proposal flux distribution, *flux*_p_, was generated by addition of random values. The *flux*_p_ was accepted at an acceptance probability *p* = *P*(RSS(*flux*_p_))/*P*(RSS(*flux*_j_)) where *P*(RSS(*flux*)) follows a χ^2^ distribution. The procedure was repeated 5,000,000 times. A population of metabolic flux ratio was produced from the latter half of the chain. The procedure was repeatedly performed for two datasets (for example control and treated). Cohen’s effect size (*d*) was used as a quantitative measure of the magnitude of the mean difference in metabolic flux ratio between the two (control and treated) datasets.

Figure 2a shows an example of mass spectra data for the isotopomer analysis. The data were acquired from our previous study (Supplementary Table S2)^40^. MCF-7 breast cancer cells that were untreated or treated with 10 nM paclitaxel were cultured in a medium containing non-labeled glucose and [U-^13^C]glutamine. The intracellular metabolites were extracted at 24 h and analyzed by mass spectrometry to determine the labeling patterns of Cit under the metabolic and isotopically steady states. The standard deviation of the measurement (σ) was previously determined to be approximately 0.015.

The isotopomer analysis usually focuses on the intensity of m4 and m5 signals of Cit ([Cit_m4_] and [Cit_m5_], respectively) (Fig. 2a). The comparison of the mass spectra showed that the intensity of [Cit_m5_] relative to [Cit_m4_] in the paclitaxel-treated cells ([Cit_m5_]/[Cit_m4_] = 1.22) was higher than that of the control cells ([Cit_m5_]/[Cit_m4_] = 0.86), suggesting that the relative contribution of IDH_reverse_ to Cit synthesis might be increased in the paclitaxel-treated cells (the metabolic flux levels of IDH_reverse_ and CS is denoted as [IDH_reverse_] and [CS] in this study) (Fig. 1a). Contrastingly, the isotope labeling pattern of Cit could also be affected by other factors. For instance, the relative abundances of both [Cit_m4_] and [Cit_m5_] decreased in the paclitaxel-treated cells. Moreover, other isotopomers, including Cit_m1_, Cit_m2_, Cit_m3_, and Cit_m6_, were observed in the measured data (Fig. 2a). Interpretation of the results of the isotope labeling experiment is not straightforward, as there is a non-linear relationship between the measured mass spectra data and metabolic flux ratio, as mentioned above.

To address this issue, a computational method was developed by an introduction of a metabolic model (Fig. 2b) and the Metropolis-Hastings algorithm (Fig. 2c). First, a metabolic model of the central carbon metabolism of cancer cells, including stoichiometry of 53 reactions, was adapted from a previous ^13^C-MFA study (Fig. 2b and Supplementary Table S3)^40^. The metabolic model considers glucose and glutamine carbon sources (In the case of the example data shown in Fig. 2a, non-labeled glucose and [U-^13^C]glutamine were used). The metabolic model could be considered as a function, *Model*, to simulate MDV of metabolites, *M*_*sim*_, calculated for a given metabolic flux distribution, *flux*: *M*_*sim*_ = *Model*(*flux*). A residual sum of square *RSS*(*flux*) between the simulated and the experimentally measured MDV (*M*_*exp*_) was also calculated (see Methods for details).

In the ^13^C-MFA, a metabolic flux distribution was estimated by searching the metabolic flux distribution, *flux*, that minimized RSS(*flux*). For the point estimation of the metabolic flux distribution, an over-determined system is essential. This means that number of data points in measured isotope labeling data have to be larger than the number of independent flux of the metabolic model (Requirement 4 in Table 1). For instance, the number of independent flux of the metabolic model shown in Fig. 2b is 23^38^.

On the other hand, measured MDV data with smaller data points have usually been used for isotopomer analyses. For example, the data point of *M*_exp_ of Cit was six, because one degree of freedom (df) was used for the data scaling. The under-determined system is unsuitable for point estimation of one metabolic flux distribution. For instance, the best-fitted *flux* could be searched for the MDV data of Cit obtained from the control sample (including six data points from Cit_m0_ to Cit_m5_) (Fig. 2a). The minimum RSS(*flux*) was close to zero (0.012). This was an over-fitting result obtained using an under-determined system. It was because the RSS was too small by the two-sized χ^2^ test (RSS(*flux*) < χ^2^_0.05_ (df = 6)) when considering that *M*_exp_ includes the measurement error derived from the analysis (σ = 0.015).

### Introduction of the Metropolis-Hastings algorithm

The measured *M*_exp_ of Cit from the control cells can be considered as an additional data to estimate *flux*. This means that a posterior distribution of *flux* under the condition that an MDV data was observed could be estimated. Since the observed *M*_exp_ includes a random experimental error following the normal distribution, a probability distribution of *flux*, *P*(RSS(*flux*)), follows a χ^2^ distribution (df = number of measurement). Here, the Metropolis-Hastings method was introduced to estimate a distribution or possible solution space of *flux* (Fig. 2c)^41^. The Metropolis-Hastings algorithm is one of the most popular Markov Chain Monte Carlo (MCMC) algorithms^42^. Based on a flux distribution at step j, *flux*_j_, a proposal flux distribution, *flux*_p_, was generated by addition of random value to *flux*_j_. If an acceptance probability *p* = *P*(RSS(*flux*_p_))/*P*(RSS(*flux*_i_)) was larger than 1.0, the *flux*_p_ was accepted as *flux*_j+1_. If *p* < 1.0, *flux*_p_ was accepted with probability *p*. When the *flux*_p_ was rejected, *flux*_j_ was also used as *flux*_j+1_ (See *Methods* for detailed procedure) Like other MCMC methods, the Metropolis-Hastings algorithm is used to generate a chain of *flux* from a sequence of probability distributions that converge to a given target distribution. In this study, the Metropolis-Hastings algorithm was repeated 5,000,000 times (approximately 130 min by Intel Core i7, 3.0 GHz to produce one chain). The data of the initial 2,500,000 steps were discarded as the burn-in process. A set of 2,500 data points sampled every 1000 steps of the latter half of the chain was used as a sample population of *flux* reflecting the additional constraint. We used the 2,500 data points derived from latter 2,500,000 steps, because at least 100,000-1,000,000 steps were required for obtaining a stable estimate (Supplementary Fig. S1).

**Figure 3:**
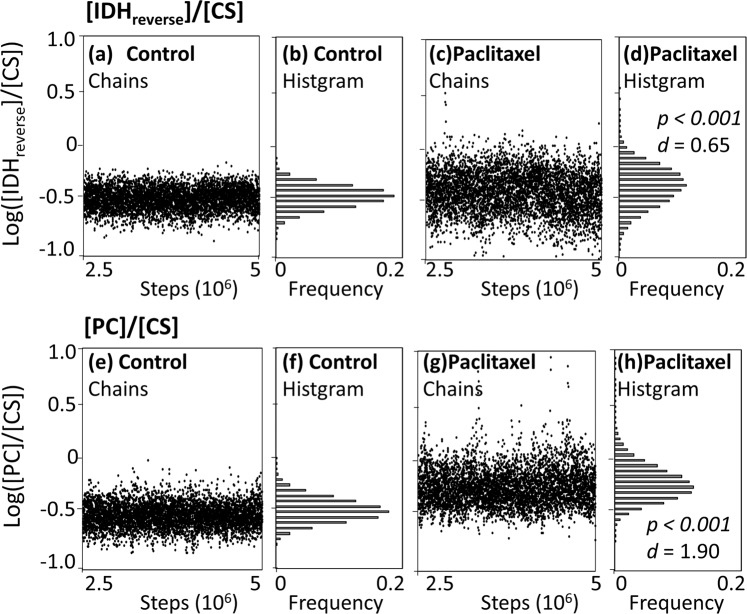
Solution flux spaces estimated from the isotope labeling data of Cit using the Metropolis-Hastings algorithm. MDV data of Cit including six data points from Cit_m0_ to Cit_m5_ were used. (**a**,**c**,**e**,**g**) Chains of log([IDH_reverse_]/[CS]) and log([PC]/[CS]) values produced by the Metropolis-Hastings method. The data of initial 2,500,000 steps were discarded as the burn-in process. (**b**,**d**,**f**,**h**) Histograms of the populations of log([IDH_reverse_]/[CS]) and log([PC]/[CS]) values produced by the Metropolis-Hastings method. The two populations produced from the isotope labeling data of Cit of control and paclitaxel-treated MCF7 cells were compared by the *p*-value from Student’s *t-*test and the Cohen’s effect size (*d*). The results are shown in panels (d,h).

For the MDV data of Cit obtained from control and paclitaxel-treated samples (Fig. 2a), two populations of *flux* were generated by the Metropolis-Hastings method (Fig. 2c). Here, a relative metabolic flux level of IDH_reverse_ to CS was compared between the control and paclitaxel-treated samples as an example. Two populations of the flux ratio, log([IDH_reverse_]/[CS]), were generated for control and paclitaxel-treated samples, respectively (Fig. 3a–d). Flux ratio values were used for the comparison, since a flux ratio between two metabolic reactions is dimensionless and comparable between two metabolic states. The populations of the control cells showed that the values of log([IDH_reverse_]/[CS]) were evenly distributed across the chain of 2,500,000 sequences (Fig. 3a). The results suggested that enough amount of samples were obtained by the Metropolis-Hastings method. The histogram shown in Fig. 3b is an estimated distributions of log([IDH_reverse_]/[CS]) values in the control cells. The procedure was also performed using the MDV data of Cit obtained from the paclitaxel-treated cells (Fig. 3c,d).

### Comparison by the Cohen’s effect size

Comparison between the two distributions showed that the mean log([IDH_reverse_]/[CS]) for the population of paclitaxel-treated data (Fig. 3d) looked larger than that of control (Fig. 3b), as the *p*-value from the Student’s t-test was *p* < 0.001. However, the small *p*-value was an over-estimation of significance due to the effect derived from the large sample size. Instead of the t-test, Cohen’s effect size (*d*) was used here. The Cohen’s effect size is a quantitative measure of the magnitude of mean difference (See Method for details)^43^. Based on the Cohen’s original criteria for mean difference, effect sizes with larger than 0.8 or less than −0.8 (|*d*| > 0.8) were considered to be large in this study^43^.

Cohen’s *d*-value between two distributions was determined to be *d* = 0.65. Based on the Cohen’s criteria, the result indicates that the measured data of *M*_exp_ of Cit (Fig. 2a) did not have a large effect to discriminate the flux ratio, log([IDH_reverse_]/[CS]), between the control and paclitaxel-treated cells. Thus, the measured data of Cit provided poor evidence to support the notion that the flux ratio, log([IDH_reverse_]/[CS]), was changed by the paclitaxel treatment.

A similar procedure was used for log([PC]/[CS]) (Fig. 3e–h). A larger mean log([PC]/[CS]) was observed for the population of the paclitaxel-treated data, with *p* < 0.001 and *d* = 1.90 (Fig. 3f,h). Since Cohen’s effect size *d* was much larger than 0.8, it can be claimed that log([PC]/[CS]) increased in the paclitaxel treated cells based on the measured data of Cit shown in Fig. 2a. These results showed a good coincidence with the metabolic flux distribution determined by the ^13^C-MFA conducted in the previous study^40^.

Estimated distribution *flux* and the Cohen’s effect size (*d*) should depend on the number of data points used for the analysis. An additional analysis for log([PC]/[CS]) showed that *d*-values were determined to be *d* = 1.67 (when using Cit m0,1,2,4,5), *d* = 1.40 (Cit m0,4,5), and *d* = 0.20 (Cit m4,5), respectively (data not shown). Level of *p-*values were less than 0.001 for all conditions. These results indicated that an MDV dataset that includes a larger number of data points is preferable to produce a population with smaller variation by the Metropolis-Hastings algorithm. To perform the Metropolis-Hastings algorithm, however, it has to be verified that the metabolic model overfits to the measured MDV data (RSS(*flux*) < χ^2^_0.05_). It indicates that an arbitrary selection of suitable MDV dataset is needed to satisfy the conditions before performing the analysis.

It should be also noted that the developed method compared flux ratios between two metabolic states, indicating that estimated *d*-values depends on denominator reactions. For instance, *d*-values of log([PC]/[CS]), log([PC]/[ACLY (ATP citrate lyase)]), and log([PC]/[Glucose uptake]) were determined to be 1.90 (Fig. 3), 2.02, and 1.74, respectively (data not shown). The variation in *d*-values suggested that a careful selection of denominator reaction is needed to perform useful isotopomer analysis.

### Reanalysis of literature reported data

**Figure 4:**
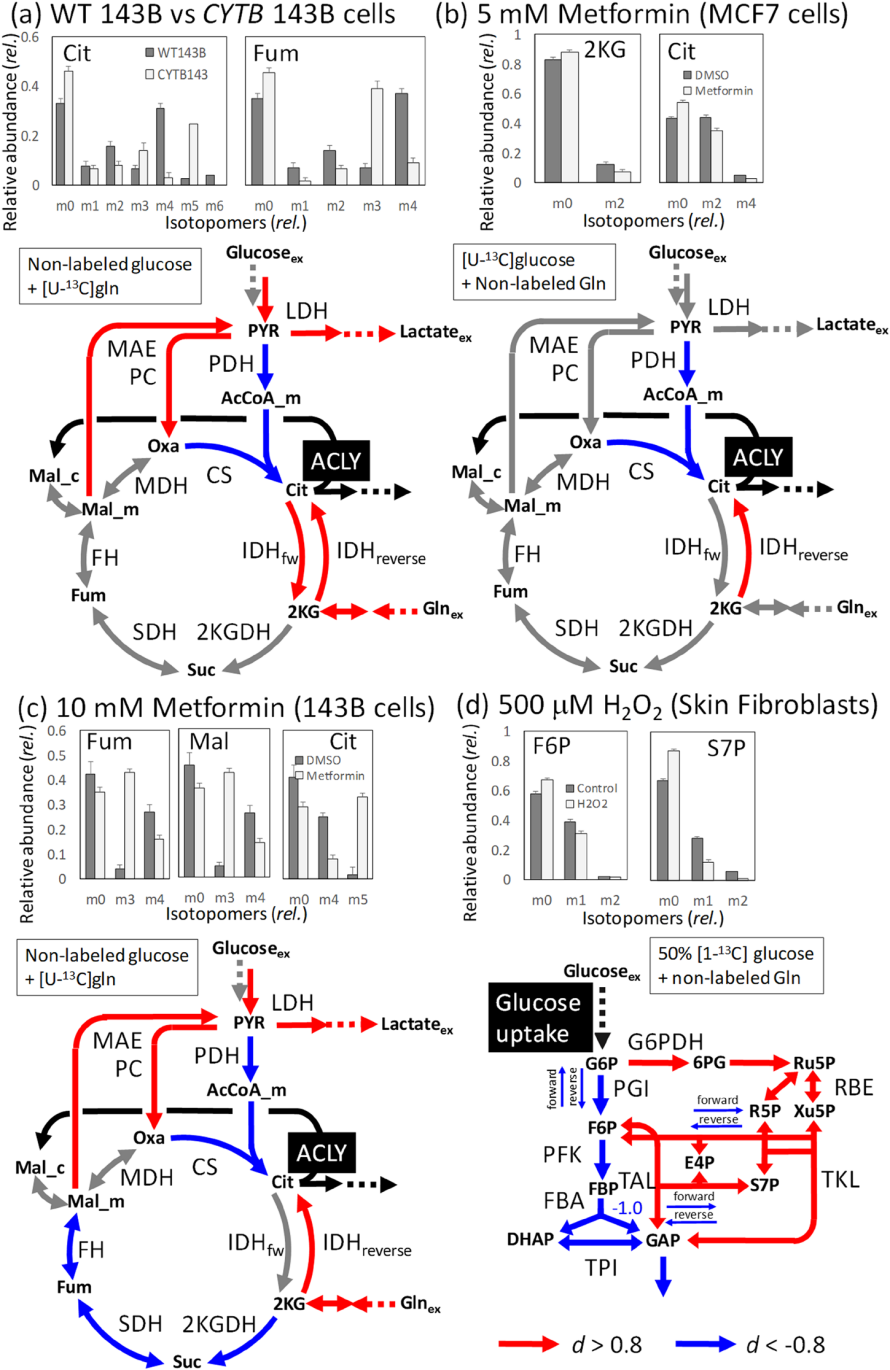
Reanalysis of the literature reported data by the Metropolis-Hastings algorithm-based method. The mass isotopomer distribution vector (MDV) data were obtained from the four isotopomer analyses reported in the literature. The results are shown in the simplified metabolic network. For a metabolic reaction X, a population of flux ratio, log([X]/[ACLY])(panels (a–c)) or log([X]/[Glucose uptake]) (for panel (d)) were obtained by the Metropolis-Hastings algorithm and compared using the Cohen’s *d*-value between two metabolic states. The denominator reactions were highlighted in black. Reactions highlighted by red and blue colors indicate the metabolic reaction whose Cohen’s *d*-values were more than 0.8 and less than −0.8, respectively. Carbon sources are shown. (**a**) Comparison between wild type (WT) 143B and a mutant (*CYTB*) 143B lacking the complex III in the electron transport chain. The MDV data was obtained from Mullen *et al*.^32^. (**b**) Comparison between the MCF7 cells non-treated and treated with 5 mM metformin reported by Andrzejewski *et al*.^33^. (**c**) Comparison between the143B cells non-treated and treated with 10 mM metformin reported by Mullen *et al*.^32^. (**d**) Metabolic redirection of human skin fibroblast induced by the treatment with 50 μM hydrogen peroxide (H_2_O_2_). The MDV data was obtained from Kuehne *et al*.^44^.

The isotopomer analysis reported in literature were reanalyzed by the method developed in this study^32,33,44^. Mullen *et al*. (2011) compared MDVs of Cit and Fum obtained from the wild type (WT) 143B cells cultured with [U-^13^C]Gln with that obtained from the mutant (*CYTB*) 143B cells lacking the complex III in the electron transport chain (Fig. 4a, bar graphs)^32^. The authors reported that glutamine was used as the major anaplerotic precursor in WT143B cells that produced Cit_m4_ from [U-^13^C]Gln. In contrast, *CYTB* 143B cells mainly produced Cit_m5_ through the reductive glutamine metabolism (Fig. 1a). MDV data obtained from these figures were presently reanalyzed by the method developed in this study (Supplementary Table S2). Two populations of *flux* were generated from the MDVs of WT and *CYTB* 143B cells by the Metropolis-Hastings method described above. For example, the flux ratio of log([CS]/[ACLY]) in *CYTB* 143B cells were smaller than that of WT 143B cells, since the *d*-value was smaller than −0.8. (*d* = −6.1, highlighted by the blue color in the lower panel of Fig. 4a; all results are shown in Supplementary Table S4). Here the metabolic flux levels of ATP:acetyl CoA lyase ([ACLY]) was used as a denominator reaction to compare relative contribution of CS and IDH_reverse_ to the citrate biosynthesis (highlighted by the black color in Fig. 4a). On the other hand, the *CYTB* 143B cells had greater flux ratio of log([IDH_reverse_]/[ACLY]) than that in WT 143B cells (*d*-value = 5.5; highlighted by the red color in Fig. 4a). These results confirmed the measured MDV data supported the activation of the reductive glutamine metabolism in *CYTB* 143B as reported in the original literature. Moreover, additional metabolic reprogramming was read out from the reanalysis. As shown in Fig. 4a, the results suggested that flux ratios, such as log([PC]/[ACLY]), log([MAE]/[ACLY]), log([IDH_fw_]/[ACLY]), and log([Gln uptake]/[ACLY]), increased in *CYTB* 143B with Cohen’s *d-*values larger than 0.8.

Metabolic reprogramming induced by the metformin treatment has been reported in the two independent studies using MCF-7 cells (Fig. 4b)^33^ and 143B cells (Fig. 4c)^32^. Metformin is a prescription drug used to treat type 2 diabetes. Andrzejewski *et al*. investigated metformin-induced metabolic reprogramming in MCF-7 cells using [U-^13^C]glucose^33^. The authors described that the metformin treatment decreased the labeling of Cit_m2_ and [^13^C_2_]2-ketoglutarate (2KG_m2_) derived from glucose, indicating that less glucose entered the mitochondrial metabolism in cells (Fig. 4b, bar graphs). The present reanalysis of reported MDV data (including 2KG_m0,m2_ and Cit_m0,m2,m4_) confirmed that the MDV data were enough to support a decrease in the flux ratio of log([CS]/[ACLY]) by the metformin treatment (*d*-value = −1.2). Moreover, it was also suggested from the MDV data that the reductive glutamine metabolism was also activated, since the *d-*value at 1.1 was observed for log([IDH_reverse_]/[ACLY]). Since similar labeling patterns of Cit and 2KG were observed between the non-treated and treated cells, the *d*-values close to the threshold level were obtained.

Similar metabolic reprogramming was observed in the isotopomer analysis of 143B cells using [U-^13^C]Gln (Fig. 4c). In the original report, only the metformin-induced activation of reductive glutamine metabolism has been mentioned based on the MDV data of Cit, Fum, and Mal (Fig. 4c, bar graph)^32^. On the other hand, the reanalysis using the Metropolis-Hastings method found that the MDV dataset could suggest an increase in log([PC]/[ACLY]), log([MAE]/[ACLY]), and log([LDH]/[ACLY]), as well as a decrease in log([CS]/[ACLY]) and log([SDH]/[ACLY]) (See Supplementary Table S4 for full results).

Figure 4d depicts the result of isotopomer analysis of human skin fibroblasts that were untreated or treated with 500 μM hydrogen peroxide cultured in medium containing 50% non-labeled and 50% [1-^13^C]glucose^44^. The authors pointed out that carbon flux was redirected from the EMPP to the oxPPP upon oxidative stress due to an increased [F6P_m0_] and [S7P_m0_] coupled to a decreased [F6P_m1_] and [S7P_m1_] fractions. The finding was presently confirmed by the reanalysis using the Metropolis-Hastings algorithm (Fig. 4d). The reanalysis also revealed a decreased metabolic flux levels of the forward and reverse reactions of PGI, TAL, and TKL suggesting an increase in the chemical motive force or a decrease in the Gibb’s free energy change (Δ*G*’) of these reactions upon the hydrogen peroxide stress.

## Discussion

Isotopomer analysis by stable isotope labeling has been widely adopted for the reliable estimation of metabolic redirection or reprogramming (Fig. 1)^32,45–47^. In this study, we developed a computational method for the analysis of the MDV data produced by the isotopomer analysis (Fig. 2c). The reanalysis of the literature data demonstrated that more detailed metabolic redirection could be revealed by the developed method (Figs. 3 and 4). A drawback of the methodology is a longer calculation time for the Metropolis-Hastings algorithm. Moreover, caution is needed when interpreting the results, since changes in metabolic flux ratio instead of metabolic flux level were evaluated. In addition, estimated metabolic redirection should be validated by additional data or experiment since these analyses were performed using a small number of MDV data and the metabolic model with several simplifications (Fig. 2). Furthermore, whereas the developed method is able to evaluate change in a flux ratio between two reactions, it is unsuitable for find a drastic flux re-organizations via novel metabolic pathways. Additional data or the ^13^C-MFA would be needed to measure a proportional increase and decrease in two metabolic flux levels.

An advantage of the developed method is that it enables an analysis of metabolic redirection using numerous data point. By the conventional method, in the case of the example shown in Fig. 4a, only a relative contribution of the reductive glutamine metabolism and forward reaction of the TCA cycle to synthesize Cit was estimated from the two data points, including [Cit_m4_] and [Cit_m5_] (Fig. 1a). The developed method was able to assess metabolic redirection in the metabolic network using the six plus four data points of Cit and Fum (Fig. 4a).

Another advantage is a quantitative assessment of more detailed metabolic redirections by Cohen’s effect size (*d*) (Figs. 3 and 4). We considered that the MDV data supported a metabolic redirection of interest when large Cohen’s effect size was observed (|*d*| > 0.8). In the case of the example shown in Figs. 2 and 3, although distinct labeling patterns were observed for [Cit_m4_] and [Cit_m5_], the reanalysis pointed out that the data was not enough to support a shift of a flux ratio between control and paclitaxel-treated MCF-7 cells, since *d* = 0.65. Notably, a larger effect size means stronger support from the measured MDV data, but does not always represent a larger shift in the metabolic ratio.

The reanalysis of the literature data also revealed that flux ratio levels, such as log([PC]/[ACLY]) and log([MAE]/[ACLY]), were increased by the metformin treatment (Fig. 4a,c). This was because the activation of these anaplerotic reactions was required to produce an amount of non-labeled form of Cit (Cit_m0_) observed in the MDV datasets. These results demonstrate that a quantitative assessment by Cohen’s effect size (*d*) enables a more detailed evaluation of metabolic reprograming by the isotopomer analysis, that should be validated by additional data or experiment. This technique will support a future application of the isotopomer analysis for various targets including cultured cells, whole tissues, and organs as well as using an advanced experimental technique such as a parallel labelling^29,48,49^.

## Methods

### Metabolic models

A metabolic model of *Homo sapiens* that is similar to that used in the previous ^13^C-MFA studies was employed^50–54^. The model includes 53 reactions and 34 metabolites in the pathways for glycolysis, pentose phosphate, TCA cycle, anaplerosis, and lipid biosynthesis (Supplementary Table S3). In the model, intracellular compartmentalization between the cytosol and mitochondria was ignored for pyruvate, citrate, and 2-ketoglutarate (2KG), based on a similar simplification process followed in previous ^13^C-MFA studies^50,52,54,55^. The following processes were assumed for the analysis: glucose uptake, glutamine uptake, production of lactate, and acetyl-CoA (AcCoA) supply required for the lipid biosynthesis. The specific glucose uptake rate was arbitrarily set to 100. Other specific rates were considered to be in free flux, with specified lower and upper thresholds, including glutamine uptake (5–50), and lactate production (50–300). The lower and upper thresholds were set arbitrarily based on the previous reports^50,52,54,55^. Uptake of other amino acids and the carbon supply for the synthesis of other building blocks were ignored in this study for simplification.

### Data source and data analysis

The ^13^C-labeling pattern data reported in the previous isotopomer analysis studies were used. MDV data including relative signal intensities and their standard deviations were measured from figures obtained from the literature (Supplementary Table S2). The minimum standard deviation of the measurement was set to 0.015.

All data analyses were performed using mfapy 0.5.2 (a Python version of OpenMebius^56^) implemented in Python 3.6 (https://github.com/fumiomatsuda/mfapy). The aforementioned metabolic model could be considered as a function, *Model*, to simulate MDV of ith measured metabolites, *M*_*i,sim*_, calculated for a given metabolic flux distribution, *flux*, by the EMU method as follows:

$${M}_{{i},{sim}}={Model}(\,{flux})$$

A residual sum of square (RSS) between the simulated and the experimentally measured MDV (*M*_*i,exp*_) as follows:

1 $$RSS(Flux)=\sum _{i}\,{(\frac{{{\rm{M}}}_{i,\exp }-{{\rm{M}}}_{i,{\rm{sim}}}}{{{\rm{\sigma }}}_{i}})}^{2}$$

where σ represents the standard deviation of measurement and RSS(*Flux*) is a RSS for a given *flux*. The Metropolis-Hastings algorithm was performed by the following procedure,

1. A seed metabolic flux distribution *flux* was produced by minimizing the RSS(*flux*) using the sequential least squares programming (SLSQP) function implemented in SciPy 1.3^57^.
2. A seed metabolic flux distribution was accepted as a 0^th^ metabolic flux distribution, *flux*_0_, if an over-fitting result with RSS(*flux*_0_) < χ^2^_0.05_ (number of measurements) was obtained. Degree of freedom was the number of measurement.
3. Based on a flux distribution at j^th^ step, *flux*_j_, a proposal flux distribution, *flux*_p_, was generated by addition of random values to flux level of three randomly selected reactions. Proposal flux distributions were iteratively generated until a *flux*_p_ within feasible flux space was obtained.
4. If an acceptance probability *p* = *P*(RSS(*flux*_p_))/*P*(RSS(*flux*_j_)) was larger than 1.0, the *flux*_p_ was accepted as *flux*_j+1_. *P* is a probability distribution of the chi-square distribution (degree of freedom was a number of measurement). If *p* < 1.0, *flux*_p_ was accepted with probability *p*. When the *flux*_p_ was rejected, *flux*_j_ was also used as *flux*_j+1_.
5. The procedure was repeated 5,000,000 times to generate a Malkov chain. The data of the initial 2,500,000 steps were discarded as the burn-in process.
6. From the population of the latter half of the chain (2,500,000 data points), 2500 *flux*_j_ were obtained by a sampling of every 1000 step of the chain.
7. The whole procedure was repeated eight times.
8. Sample population including 20,000 *flux*_j_ (2500 with eight replicates) were used for the following data analysis. The time required for the Metropolis-Hastings algorithm was approximately 130 min by Intel Core i7, 3.0 GHz per one dataset.

Two populations were generated, such as for conditions A and B, in terms of a flux ratio, [M]_i,n_/[L]_i,n_, where, [M]_i,n_ and [L]_i,n_ indicate the metabolic flux levels of reactions M and L under the condition *i* determined by the *n*th sample. The Cohen’s effect size (*d*) was determined as follows^58^:

$$d=(\bar{A}-\bar{B})/\sqrt{(({n}_{A}-1){s}_{A}^{2}+({n}_{B}-1){s}_{B}^{2})/({n}_{A}+{n}_{B}-2)}$$

where $\bar{A}$ and $\bar{B}$ are the means of populations A ([M]_A,n_/[L]_A,n_) and B ([M]_B,n_/[L]_B,n_). *s*_A_ and *s*_B_ are the standard deviations, and *n*_A_ and *n*_B_ are the sizes of populations A and B. Based on the Cohen’s original criteria for the case of mean difference, effect sizes larger than 0.8 or less than −0.8 (|*d*| > 0.8) were considered large.

## Supplementary information

supplementary information.
