# Supplementary material for: Computational data mining method for isotopomer analysis in the quantitative assessment of metabolic reprogramming
Source: Sci Rep. 2020 Jan 14;10:286. doi: 10.1038/s41598-019-57146-8 (PMC6959353; doi:10.1038/s41598-019-57146-8)
Supplement: Supplementary file 1 — supplementary information. [file 41598_2019_57146_MOESM1_ESM.pdf]

Supplementary Figure S1 Relationship between the population size and Cohen's effect size (*d*). Cohen's effect sizes (*d*) were determined for log([IDHreverse]/[CS]) and log([PC]/[CS]) of the populations, at different population sizes.

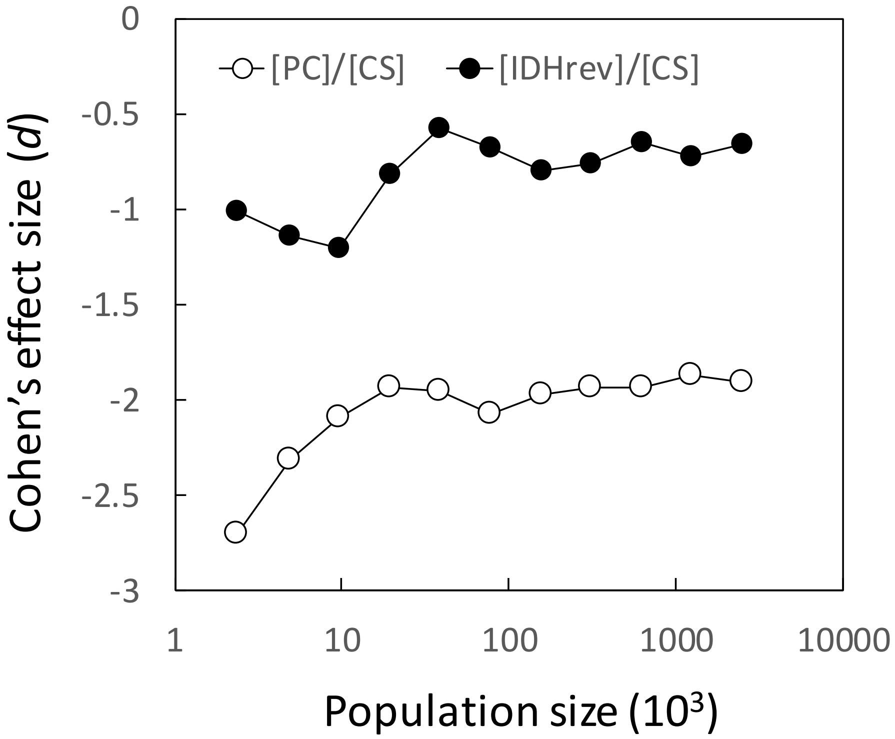

Supplementary Table S1 Abbreviations of metabolites and reactions

PGI	Phoshoglucoisomerase	G6P<->F6P
PFK	Phoshofruktokinase	F6P->FBP
FBA	Aldolase	FBP<->DHAP+GAP
TPI	Triose isomerase	DHAP<->GAP
PGK	Phosphoglycerate kinase	GAP<->PGA
ENO	Enolase	PGA<->PEP
PYK	Pyruvate kinase	PEP->Pyr
PDH	Pyruvate dehydrogenase	Pyr->AcCOA+CO2
CS	Citrate synthase	AcCOA+Oxa->IsoCit
IDH	Isocitrate dehydrogenase	IsoCit->aKG+CO2
2KGDH	2-Ketoglutarate dehydrogenase	aKG->Suc+CO2
SDH	Succinate dehydrogenase	Suc<->Fum
FH	Fumarase	Fum<->Mal
MDH	Malate dehydrogenase	Mal<->Oxa
G6PDH	Glucose-6-phosphate dehydrogenase	G6P->6PG
6PGDH	6-phosphogluconate dehydrogenase	m6PG->Ru5P+CO2
RPE	Ribulose-5-phosphate epimerase	Ru5P<->R5P
RPI	Ribose-5-phosphate isomerase	RU5P<->Xu5P
TKL1	Transketolase	E4P+Xu5P<->F6P+GAP
TKL2	Transketolase	R5P+Xu5P<->S7P+GAP
TAL	Transaldolase	GAP+S7P<->F6P+E4P
MAE	Malic enzyme	Mal->Pyr+CO2
PC	Pyruvate carboxylase	Pyr+CO2->Oxa
ACLY	ATP citrate lyase	IsoCit+AcCOA->Mal+Suc
LDH	Lactate dehydrogenase	Pyr -> Lactate
G6P	Glucose-6-phosphate	
F6P	Fructose 6-phosphate	
FBP	Fructose-1,6-bisphosphate	
DHAP	Dihydroxyacetone phosphate	
GAP	Glyceraldehyde 3-phosphate	
6PG	6-Phosphogluconate	
Ru5P	Ribulose 5-phosphate	
R5P	Ribose 5-phosphate	

S7P	Sedoheptulose 7-phosphate
E4P	Erythrose 4-phosphate
Xu5P	Xylulose 5-phosphate
3PG	3-Phosphoglycerate
PEP	Phosphoenolpyruvate
Pyr	Pyruvate
AcCoA	Acetyl-CoA
Cit	Citrate
IsoCit	Isocitrate
2KG	2-ketoglutaric acid
Suc	Succinate
Fum	Fumarate
Mal	Malate
Oxa	Oxaloacetic acid
Gln	Glutamine
EMPP	Embden–Meyerhof–Parnas pathway
oxPPP	oxidative pentose phosphate pathway
Glu	Glutamate

Supplementary Table S2. Mass isotopomer distribution vector (MDV) data obtained from literatures

Metabolite	Isotopomer	Intensity (rel.)	Standard deviation
Araki et al (2017) MCF-7 control			
Cit	m0	0.319	0.015
Cit	m1	0.039	0.015
Cit	m2	0.104	0.015
Cit	m3	0.170	0.015
Cit	m4	0.186	0.015
Cit	m5	0.160	0.015
Araki et al (2017) MCF-7 paclitaxel treated			
Cit	m0	0.511	0.015
Cit	m1	0.069	0.015
Cit	m2	0.087	0.015
Cit	m3	0.112	0.015
Cit	m4	0.098	0.015
Cit	m5	0.120	0.015
Muller et al (2012) Wild type 143B			
Fum	m0	0.35	0.02
Fum	m1	0.07	0.02
Fum	m2	0.14	0.02
Fum	m3	7.00E-02	0.015
Cit	m0	0.33	0.015
Cit	m1	0.075	0.015
Cit	m2	0.155	0.015
Cit	m3	0.065	0.015
Cit	m4	0.31	0.015
Cit	m5	0.025	0.015
Mullen et al (2012) CYTB 143B			
Fum	m0	0.455	0.02
Fum	m1	0.015	0.015
Fum	m2	0.065	0.015
Fum	m3	0.39	0.03
Cit	m0	0.46	0.07
Cit	m1	0.065	0.03
Cit	m2	0.08	0.04
Cit	m3	0.14	0.02
Cit	m4	0.03	0.04
Cit	m5	0.245	0.045
Andrzejewski et al (2014) MCF7 control			
Cit	m0	0.43	0.015
Cit	m2	0.44	0.015

Cit	m4	0.05	0.015
2KG	m0	0.83	0.015
2KG	m2	0.12	0.015
Andrzejewski et al (2014) MCF7 5 mM metformin treated			
Cit	m0	0.54	0.015
Cit	m2	0.35	0.015
Cit	m4	0.025	0.015
2KG	m0	0.88	0.015
2KG	m2	0.07	0.015
Mullen et al (2012) 143B control (DMSO)			
Fum	m0	0.425	0.05
Fum	m3	0.04	0.015
Fum	m4	0.27	0.03
Cit	m0	0.41	0.03
Cit	m4	0.25	0.03
Cit	m5	0.015	0.01
Mal	m0	0.455	0.05
Mal	m3	0.045	0.015
Mal	m4	2.60E-01	0.02
Mullen et al (2012) 143B 10 mM metformin treated			
Fum	m0	0.35	0.02
Fum	m3	0.43	0.015
Fum	m4	0.16	0.015
Cit	m0	0.29	0.015
Cit	m4	0.08	0.03
Cit	m5	0.33	0.04
Mal	m0	0.36	0.02
Mal	m3	0.425	0.02
Mal	m4	1.40E-01	0.015

Supplementary Table S3. Metabolic model used in this study.

Reaction ID	Stoichiometry	Atom mapping	Fixed/ free	Lower boundary	Upper boundary
r1_SubsGlc	SubsGlc --> G6P	ABCDEF --> ABCDEF	fixed	100	100
r2_pgi	G6P --> F6P	ABCDEF --> ABCDEF	free	0.0001	1000
r3_pgi	F6P --> G6P	ABCDEF --> ABCDEF	free	0.0001	1000
r4_pfk	F6P --> FBP	ABCDEF --> ABCDEF	free	0.0001	1000
r5_fba	FBP --> DHAP + GAP	ABCDEF --> CBA + DEF	free	0.0001	1000
r6_fba	DHAP + GAP --> FBP	CBA + DEF --> ABCDEF	free	0.0001	1000
r7_tpi	DHAP --> GAP	ABC --> ABC	free	0.0001	1000
r8_tpi	GAP --> DHAP	ABC --> ABC	free	500	1000
r9_gapdh	GAP --> PGA	ABC --> ABC	free	0.0001	1000
r10_gapdh	PGA --> GAP	ABC --> ABC	free	0.0001	1000
r11_peph	PGA --> PEP	ABC --> ABC	free	0.0001	1000
r12_peph	PEP --> PGA	ABC --> ABC	free	0.0001	1000
r13_pk	PEP --> Pyr	ABC --> ABC	free	0.0001	1000
r16_pyrdh	Pyr --> AcCOAmit + CO2in	ABC --> BC + A	free	0.0001	1000
r17_cs	AcCOAmit + Oxa --> Cit	AB + CDEF --> FEDBAC	free	0.0001	1000
r18_idh	Cit --> aKG + CO2in	ABCDEF --> ABCDE + F	free	0.0001	1000
r19_idh	aKG + CO2in --> Cit	ABCDE + F --> ABCDEF	free	0.0001	1000
r20_akgdh	aKG --> Suc + CO2in	ABCDE --> BCDE + A	free	0.0001	1000
r21_sdh	Suc --> Fum	ABCD --> ABCD	free	0.0001	1000
r22_sdh	Fum --> Suc	ABCD --> ABCD	free	0.0001	1000
r23_fh	Fum --> Mal	ABCD --> ABCD	free	0.0001	1000
r24_fh	Mal --> Fum	ABCD --> ABCD	free	0.0001	1000
r25_mdh	Mal --> Oxa	ABCD --> ABCD	free	0.0001	1000
r26_mdh	Oxa --> Mal	ABCD --> ABCD	free	0.0001	1000
r27_pc	Pyr + CO2in --> Oxa	ABC + D --> ABCD	free	0.0001	1000
r28_mae	Mal --> Pyr + CO2in	ABCD --> ABC + D	free	0.0001	1000
r29_g6pdh	G6P --> m6PG	ABCDEF --> ABCDEF	free	0.0001	1000
r30_pgd	m6PG --> Ru5P + CO2in	ABCDEF --> BCDEF + A	free	0.0001	1000
r31_ri	Ru5P --> R5P	ABCDE --> ABCDE	free	0.0001	1000
r32_ri	R5P --> Ru5P	ABCDE --> ABCDE	free	0.0001	1000
r33_rbe	Ru5P --> Xu5P	ABCDE --> ABCDE	free	0.0001	1000
r34_rbe	Xu5P --> Ru5P	ABCDE --> ABCDE	free	0.0001	1000
r35_tkt	R5P + Xu5P --> S7P + GAP	ABCDE + FGHIJ --> FGABCDE + HIJ	free	0.0001	1000
r36_tkt	GAP + S7P --> Xu5P + R5P	HIJ + FGABCDE --> FGHIJ + ABCDE	free	0.0001	1000
r37_tal	GAP + S7P --> F6P + E4P	ABC + DEFGHIJ --> DEFABC + GHIJ	free	0.0001	1000
r38_tal	E4P + F6P --> S7P + GAP	GHIJ + DEFABC --> DEFGHIJ + ABC	free	0.0001	1000
r39_tkt	E4P + Xu5P --> F6P + GAP	ABCD + EFGHI --> EFABCD + GHI	free	0.0001	1000
r40_tkt	GAP + F6P --> Xu5P + E4P	GHI + EFABCD --> EFGHI + ABCD	free	0.0001	1000

r41_ldh	Pyr --> Lac	ABC --> ABC	free	0.0001	1000
r42_Lac_ex	Lac --> LacEx	nd	free	50	200
r46_SubsGln	SubsGln --> Gln	ABCDE --> ABCDE	free	5	50
r47_gls	Gln --> Glu	ABCDE --> ABCDE	free	0.0001	1000
r48_gls	Glu --> Gln	ABCDE --> ABCDE	free	0.0001	1000
r49_gludh	Glu --> aKG	ABCDE --> ABCDE	free	0.0001	1000
r50_gludh	aKG --> Glu	ABCDE --> ABCDE	free	0.0001	1000
r82_acl	Cit --> AcCOAcyt + Malcyt	FEDBAC --> AB + CDEF	free	0.0001	1000
r83_Malt1	Malcyt --> Mal	ABCDE --> ABCDE	free	0.0001	1000
r83_Malt2	Mal --> Malcyt	ABCDE --> ABCDE	free	0.0001	1000
r83_AcCOA_BIO	AcCOAcyt --> AcCOABiomass	nd	free	0.0001	1000
r100_CO2	CO2in --> CO2Ex	A --> A	free	0.0001	1000
r101_CO2	SubsCO2 --> CO2in	A --> A	free	0.0001	1000

Supplementary Table S4. Reanalysis of literature reported flux ratio analysis

X	*d*-Values for comparison of log([X]/[ACLY])			*d*-Values for comparison of log([X]/[r1_SubsGlc])
	Fig. 4a: WT 143B vs CYTB 143B	Fig. 4b: 5mM Metformin (MCF7)	Fig. 4c: 10mM Metformin (143B)	Fig. 4d: 500microM H2O2 (Skin fibroblast)
r1_SubsGlc	4.34	-0.32	3.62	nd
PGI	1.72	-0.06	1.28	-0.83
r4_pfk	4.31	-0.28	3.52	-0.96
FBA	4.31	-0.28	3.52	-0.96
TPI	4.31	-0.28	3.52	-0.96
GAPDH	4.39	-0.30	3.62	-0.96
PEPH	4.39	-0.30	3.62	-0.96
r13_pk	4.39	-0.30	3.62	-0.96
r41_ldh	5.14	0.04	4.29	-0.04
r29_g6pdh	1.29	-0.32	1.47	0.94
r30_pgd	1.29	-0.32	1.47	0.94
RPI	1.29	-0.32	1.47	0.94
RBE	1.29	-0.32	1.47	0.94
TKT1	1.29	-0.32	1.47	0.94
TAL	1.29	-0.32	1.47	0.94
TKT2	1.29	-0.32	1.47	0.94
r27_pc	9.57	0.27	3.82	-0.01
r28_mae	8.67	-0.04	4.85	-0.02
r16_pyrdh	-6.09	-1.21	-4.82	-0.03
r17_cs	-6.09	-1.21	-4.82	-0.03
r20_akgdh	-0.59	-0.38	-1.24	0.04
r18_idh	3.31	-0.58	0.12	0.12
r19_idh	5.47	1.05	5.20	0.11
SDH	-0.59	-0.38	-1.24	0.04
FH	-0.59	-0.38	-1.24	0.04
r25_mdh	-0.69	-0.33	1.30	0.08
r26_mdh	-0.45	-0.20	1.34	0.09
r46_SubsGln	7.44	-0.06	5.26	0.00
GLS	7.44	-0.06	5.26	0.00

GLUDH	7.44	-0.06	5.26	0.00
r82_acl	nd	nd	nd	-0.04
r2_pgi	nd	nd	nd	-2.31
r3_pgi	nd	nd	nd	-1.80
r5_fba	nd	nd	nd	-0.13
r6_fba	nd	nd	nd	-0.10
r7_tpi	nd	nd	nd	0.21
r8_tpi	nd	nd	nd	0.24
r9_gapdh	nd	nd	nd	-0.06
r10_gapdh	nd	nd	nd	-0.04
r11_peph	nd	nd	nd	-0.08
r12_peph	nd	nd	nd	-0.07
r21_sdh	nd	nd	nd	0.12
r22_sdh	nd	nd	nd	0.11
r23_fh	nd	nd	nd	-0.08
r24_fh	nd	nd	nd	-0.09
r25_mdh	nd	nd	nd	0.08
r26_mdh	nd	nd	nd	0.09
r31_ri	nd	nd	nd	0.13
r32_ri	nd	nd	nd	0.11
r33_rbe	nd	nd	nd	-0.46
r34_rbe	nd	nd	nd	-0.56
r35_tkt	nd	nd	nd	-0.54
r36_tkt	nd	nd	nd	-0.57
r37_tal	nd	nd	nd	-1.49
r38_tal	nd	nd	nd	-2.04
r39_tkt	nd	nd	nd	-1.12
r40_tkt	nd	nd	nd	-1.58
